# Governance under the Covid-19 pandemic: comparative perspectives on Germany and Hungary: Special section “Democratic Health in the Corona Pandemic. The Corona Pandemic as a Trigger or Amplifier of Democratic Erosion?”

**DOI:** 10.1007/s12286-022-00546-4

**Published:** 2022-12-22

**Authors:** Christian Schweiger

**Affiliations:** Chair for Comparative European Governance Systems, Institute for Political Science, Faculty of Philosophy, Chemnitz University of Technology, Thüringer Weg 9, 09126 Chemnitz, Germany

**Keywords:** Germany, Hungary, Comparative politics, Governance, Deutschland, Ungarn, Vergleichende Politikwissenschaft, Regierungslehre

## Abstract

By adopting a comparative approach between different regime types, the paper concentrates on Germany and Hungary as case studies for the comparative analysis of the effects the pandemic has had on national governance in the two countries which most strongly represent the growing cleavage between the EU’s liberal Western core and the *illiberal *Central-Eastern periphery. Methodologically the analysis follows the Most Different Systems Design and examines to what extent the Covid pandemic has functioned as a potential catalyser for the weakening of democratic governance in formerly solid democratic political systems and/or as an accelerator of democratic backsliding in hybrid regimes. For this purpose, the paper examines the process and the content of legislation passed domestically to contain the effects of the pandemic. The analysis shows that even under the stronger coordination of executive decision-making between the federal and regional government level, the foundations of legislative and judicial scrutiny remained resilient during the pandemic in Germany’s multi-level polity, while in Hungary central government has used the pandemic to substantially expand its executive powers at the expense of legislative and judiciary powers.

## Abstract (de)

Das Paper konzentriert sich auf der Basis eines Vergleiches zwischen unterschiedlichen Regimetypen auf Deutschland und Ungarn als Fallstudien für die vergleichende Analyse der Auswirkungen der Pandemie auf das Regieren auf nationaler Ebene in den beiden Ländern, die die wachsende Kluft zwischen dem liberalen Kern der EU und der *illiberalen *mittelosteuropäischen Peripherie am deutlichsten repräsentieren. In methodischer Hinsicht folgt die Analyse dem *Most Different Systems Design* und untersucht in welchem Ausmaß die Covid Pandemie als potenzieller Auslöser für die Schwächung demokratischer Kontrolle in bisher gefestigten demokratischen Systemen und/oder als Beschleuniger für das Zurückschreiten im Demokratisierungsprozess in hybriden Regimen gewirkt hat. Zu diesem Zweck untersucht das Papier den Prozess und den Inhalt von Gesetzgebung, die zur Eindämmung der Pandemie auf nationaler Ebene verabschiedet wurde. Die Analyse zeigt, dass die Fundamente legislativer und judikativer Kontrolle sogar im Rahmen der verstärkten Koordinierung exekutiver Entscheidungen zwischen der regionalen und föderalen Regierungsebene im politischen Mehrebenensystem Deutschlands stabil geblieben sind, während in Ungarn die Zentralregierung die Pandemie dafür instrumentalisiert hat, ihre Exekutivmacht auf Kosten legislativer und judikativer Befugnisse deutlich zu erweitern.

## Introduction

The Covid-19 virus pandemic represents an unprecedented crisis which fundamentally challenges national and supranational governance in Europe and across the world. The analysis presented in this paper offers a comparative perspective on how the crisis has affected national governance in selected European Union member states. The choice for the comparative case studies is determined by the EU’s East-West cleavage, which has become one of the defining characteristics of the EU’s internal setup since the gradual eastward enlargement between 2004 and 2013. The supranational governance of eastward enlargement and the EU’s subsequent multiple crisis management—from the eurozone to the refugee and the current pandemic—was substantially determined by Germany who moved centre-stage as the EU’s semi-hegemon (Paterson 2011; Bulmer and Paterson 2013). In contrast the 2004 Central-Eastern European (CEE) accession group, including the late joiners Bulgaria, Romania (2007) and Croatia (2013), remains in a peripheral position in the EU. The CEE periphery is characterised by a noticeablegap in economic competitiveness, wages, living standards and ultimately the quality of democratic governance when compared to the majority of the EU-15 countries (Farkas 2018; Agh 2016).

The Covid-19 pandemic hit the EU at a time when the CEE region had already displayed the tendency towards backsliding on the consolidation of democratic domestic institutions, most prominently in the self-proclaimed illiberal democracies of Hungary and Poland. The crucial question is therefore if the pandemic has accelerated the erosion process in the fragile CEE democracies and consequently widened the distance towards the EU’s liberal democratic core further. At the same time, it is important to scrutinise potentially adverse effects the crisis has had on the functioning of democracies in the EU-15 core. The analysis in this paper concentrates on Germany and Hungary as comparative case studies for executive governance during the pandemic in the EU’s liberal core and the CEE periphery. The two countries are representative for one of the EU’s current fundamental internal cleavages between the core of member states with functioning liberal democracies and the Central-Eastern periphery, which is characterised by what The Economist Intelligence Unit classifies as “flawed democracies” in its latest Democracy Index (DI) published in 2021. The DI adopts a ranking classification system based on the quality of the electoral process and pluralism, the functioning of executive government, political participation, political culture and civil liberties. All 12 CEE EU member states are currently classified as “flawed democracies” on the basis of the assessment that “formal democratic mechanisms co-exist with a poor functioning of government” (Economist Intelligence Unit 2022, p. 43). The EIU report points out in this respect that corruption and lack of transparency is a common feature in the executive governments of the member states in the Eastern periphery (Ibid). Amongst the CEE-12 group Hungary is ranked as the country with the second lowest score for the quality of the democratic system on the DI scale, only surpassed by Romania. Hungary receives a total of 6.5 out of the total score of 10 available and scores particularly low on political participation (5.0). It is also placed in joint 56th place with Croatia in the overall global ranking of the 1967 countries examined by the index (Economist Intelligence Unit 2022, p. 44).

In stark contrast to the Hungarian case, Germany is ranked as a “full democracy” in 15th place in the DI scale and in 10th place compared to other countries in Western Europe. It scores particularly strong on electoral pluralism and civil liberties (9.58 and 9.12) but is weaker when compared to its Western European counterparts when it comes to the functioning of government (8.21), political participation (8.33) and political culture (8.13) (Economist Intelligence Unit 2022, p. 62). In this respect the analysis singles out Germany as one of example of the “trend towards political fragmentation” under the pandemic conditions in Western Europe, which has made it harder to form governing majorities, as is shown by the formation of the first three-party coalition on the federal government level in Germany since the formation of the Federal Republic in 1949 (Ibid, p. 63).

It is hence crucial to take a closer look at the political fallout from the Covid pandemic in a comparative perspective and to examine if and to what extent it has transformed the nature of comparative government in individual countries. The analysis presented here compares two cases which should be considered as cases which fit into the Most Different System Design approach in the comparative method, with Germany’s federal multi-level governance system standing in stark contrast to Hungary’s centralised and increasingly hybrid democracy. The analysis concentrates on Covid as a common crisis as the potential independent variable in systemic change towards stronger executive government at the expense of legislative and also judicial scrutiny.

Apart from single country studies, a number of comparative studies have already been published on how individual countries tried to manage the pandemic but most of them concentrate on comparisons between similar polities in the same region. Examples are the comparison between Germany, Austria and Switzerland in Western Europe (Czypionka and Reiss 2021; Hegele and Schnabel 2021), the Czech Republic, Hungary and Bulgaria in Central-Eastern Europe (Löblová, Rone and Borbáth 2021). More systematic large-scale cross-country comparisons have measured the impact on individual freedoms and concentration of executive powers (Engler et al. 2021) and on parliamentary scrutiny in the cases of France, Germany, Hungary, Italy, Switzerland, and the United Kingdom (Bolleyer and Salát 2021), with Hungary singled out as the most centralised systems amongst the selection.

## Germany and Hungary as contrasting cases of national governance

Germany and Hungary both have only limited path-dependency in the development of their domestic polities when compared to other cases such as the United Kingdom. By comparison, the current constitutions of the democratic state in Germany and Hungary have emerged on the basis of radical systemic changes, which resulted from fundamental historic tipping points. In the German case these were the downfall of the totalitarian regime of the National Socialists and the subsequent military occupation by the WW2 Allies, followed by the division of Germany into two states in 1949 and reunification in 1990. Hungary experienced four decades of communist totalitarianism between 1949 and 1989, followed by an ongoing period of post-communist transformation, which since 2004 has been taking place in the context of EU membership and more recently has witnessed substantial backtracking on maintaining the essential values of the liberal democratic state.

### Germany’s federal multi-level polity

The polity of the Federal Republic of West Germany, which was founded under the occupation of the Western World War Two allies the United States, the United Kingdom and France, represented a “semi-sovereign state”. This particular polity was not only limited in its powers by the constraints imposed by the Allied powers (Kundnani 2014, p. 24) but also by its internal setup as a federal state, based on a system of multi-level governance. Under Allied supervision, West German elites developed the Basic Law as the new draft constitution which became the basis for a multi-level system of government, where powers are dispersed between a multiplicity of different actors on the local, regional and federal levels of government under the principles of *decentralisation *and *cooperation *(Hoyer 2001, p. 89). The federal government is consequently required to seek constant consensus with various veto players, most noticeable through negotiations between the parties within the federal government coalition and in the process of co-decision-making between the national parliament *(Bundestag) *and the Federal Council (*Bundesrat*), where the regional governments are represented (Bulmer and Paterson 2019, p. 27).

The consensual nature of the German polity is reflected by the nature of the federal system, which is grounded in the principle of “cooperative federalism” (Jeffery and Rowe 2014, p. 36) under which the federal government and the Länder are part of an interlocked process of co-decision making. Fritz W. Scharpf famously characterised this as the inherent “joint decision trap” of German federalism, where “the exercise of most governing functions is shared between the federal government and the Länder governments” (Scharpf 1988, p. 243). The constitutional provisions determined in the Basic Law reflect the inherent preference for devolved government in the German polity. Article 70 GG gives the Länder the basic right to determine their own laws in areas outside the exclusive competences granted to the federal government level, which are listed in Article 73 GG and range from defence and foreign affairs, to citizenship, monetary policy and policing matters in the area of defence against terrorism and threats to the unity of the federal state. The competitive areas of German federalism, where the Länder only have the right to determine their own laws as long as the federal government does not decide to legislate, are listed in Article 74 GG, paragraph 1. These include crucial areas such as public and criminal law (number 1), labour market regulation (number 12) and higher education admission and diplomas (number 33).

Germany’s cooperative federalism is grounded in the premise that the federal government has to ensure “the equality of living conditions” across the regions, as well as the legal and economic unity of the federal state, determined in Article 72, paragraph 2 GG. This principle has however been an ongoing source of contention between the federal and the Länder governments. The formal legal constitutional provision limits the federal government to law-making in the areas listed in Article 74 GG to exceptional circumstances and considers the main responsibility for the ensuring equal living conditions across the country to rest with the regional governments by prioritising financial support in individual policy areas (Ragnitz and Thum 2019, p. 13–14). German federalism hence requires the federal government to grant substantial financial support for each of the Länder and also includes a system of fiscal redistribution (“Länderfinanzausgleich”) between economically stronger and poorer regions (Article 107 GG) (Deutscher Bundestag 2020a). The regional perspectives on this system differ substantially, with a division between advocates of a more coordinative approach and those who favour regional distinction and policy difference. The latter can be particularly found amongst the regions with a strong regional identity, most prominently in Bavaria, Lower Saxony and Thuringia (Jeffery and Pamphilis 2016, p. 188 and 190).

A crucial means to maintain the cooperative nature of German federalism has been the consultation between the regional and the federal governments in the context of the *Landesministerkonferenzen* (LMKs) and the *Ministerpräsidentenkonferenz *(MPK). The LMKs are regular issue-related consultations, which take place in various ministerial constellations, predominantly between representatives of the regional administrations and also frequently includes representatives from the federal government. The MPK is a top-level consultative mechanism between the regional heads of government on common strategic issues for the Länder, often also as a platform for the coordination of policy positions towards the federal government. As such the LMKs and the MPK represent the “third level” of voluntary policy coordination between the regions beyond their formal interaction in the legislative process in the *Bundesrat* (Hegele and Behnke 2013, p. 22–23). These have become even more important since the reunification of Germany in 1990, which increased the number of *Länder *in the unified German state from 11 to 16. At the same time the reunification process under the old Article 23 of the Basic Law (as opposed to the possibility to create a new constitution under Article 146 GG) included the five new Länder in the institutional structure of the polity which was developed by West German political elites under the supervision of the Western Allies (Holtmann 1999, p. 87). This increased the complexity of the system of vertical (i.e. federal—regional) and horizontal (i.e. inter-regional) policy coordination (Scharpf 1999, p. 2) under conditions where the range of regional interests has substantially increased, not least due to a new East-West cleavage, which made it necessary to initiate reforms to the system. The reform to the federal system initiated in 2006 deepened the interlinkage between the federal government and the Länder in decision-making terms by creating a “network of joint decisions”, while at the same time strengthening the autonomous administrative competences of the regional governments (Sturm 2013). The result has been the growing fragmentation of regional education policies in Germany, most visible in the school system, which is characterised by a patchwork of diverse structures and regulations (Nikolai 2020), with only limited success in strengthening horizontal inter-regional cooperation through institutionalisation (Scheller 2020). The Covid pandemic has raised new questions about the efficiency of the interlinked system of cooperative governance in Germany’s multi-level system of governance.

### The expanding centralised state in Hungary

The Hungarian polity represents a relatively centralised state structure with a unicameral parliament, a strong focus on executive powers on the national level with a lack of veto points on the regional level. Since Maastricht been orientated towards introducing subsidiarity and “policy empowerment” by strengthening devolved regional and local administrative powers (Bache 2015, p. 25). In Hungary the trend has gone in the opposite direction since April 2010, when Fidesz under the leadership of prime minister Viktor Orbán took power. Since 2010 Hungary has witnessed a sustained and gradual period of centralisation at the expense of cautious attempts under the preceding Socialist governments to strengthen regional administrative capabilities (Nyikos 2021, p. 257). The same applies to the Hungarian party system and the division of powers between the central institutions of the state, which seemed to develop towards a system of liberal pluralism and functioning checks and balances during the 1990s and early 2000s (Lorenz and Bos 2021, p. 8).

In addition to the obvious trend towards centralisation, under Fidesz the foundations of the democratic state has been persistently weakened. Fidesz is a right-wing populist, nationalist and eurosceptic party which was originally founded in 1988 as a radical student movement in opposition to the communist regime. The party has been substantially shaped by Viktor Orbán, a formerly liberal activist, who built up his political profile in opposition to the communist ideology and in favour of the transformation of Hungary towards a liberal democracy (Lendvai 2017, p. 25). After the fall of the iron curtain, Hungary’s first two decades of post-communist transition and in particular the immediate years following accession to the EU in 2004 were strongly influenced by the reformed Socialist Party, which became increasingly discredited as a result of mounting economic and social problems. Orbán had already once taken office as prime minister between 1998 and 2002 in a three party coalition between Fidesz, the Hungarian Democratic Forum and the United Smallholders Party. It was however only in 2010, after eight years of continuous Socialist rule, that Orbán managed to form a coalition with its satellite party KDNP, the Hungarian Christian Democratic People’s Party. 2010 became a watershed in Hungarian politics because it reflected the growing disillusionment of the Hungarian public with the post-communist transformation process, which since 2004 had been characterised by a mainly passive adaption to the process of conditional Europeanisation. EU conditionality in the economic area was characterised by the drive towards market liberalisation, which combined with the mismanagement of the social fallout from the economic transformation, had profound effects on the Hungarian economy. The formerly leading economy of the European leg of the communist Comecon trading area under the supervision of the Soviet Union, had turned into laggard with mounting economic, fiscal and social problems and ultimately the trend towards “backsliding” on the conditionality of EU membership (Pogatsa 2009). This resulted in dwindling trust in the still weak democratic system, which was widely considered as “imported institutional transformation”, i.e. prescribed externally as part of the EU membership criteria (Agh 2019, p. 75–76). In addition, the Hungarian case is one which is exemplary for what has been described as the growing feeling of “woundedness” and “victimisation” (Farkas and Mate-Toth 2018, p. 37) as a result of the process of externally guided adaptation to EU membership and liberal market capitalism. This wider trend in the Central-Eastern European region, which became particularly visible in Hungary two decades after the end of communism, manifested itself in the tendency “to make outside factors and players responsible for unsolved problems” and to create “clear demarcation lines inside and outside of particular societies” (Ibid). This situation opened the pathway for Orbán to fill the emerging political void with an populist anti-establishment agenda which brought him on course towards political victory and towards long-term political consolidation. He steered Fidesz on course for a two third governing majority at the 2010 national elections with 57.2 per cent of the votes (Foundation Robert Schuman 2010). Orbán has since maintained the electoral standing of Fidesz in all subsequent elections and defended his two thirds majority, which made a number of constitutional changes possible. The basis for this success was the reform of the electoral law in 2011, which introduced single member districts in support of the structural majority of Fidesz (Maskarinec and Charvát 2022). This enabled another decisive Fidesz win with a two-third majority even against the broad all-party opposition alliance spearheaded by Peter Marky Zay, the mayor of the Southern Hungarian city Hódmezővásárhely at the national elections on 3 April 2022 (Hungary Today 2022).

Orbán has used his governing majorities since 2010 to reshape the Hungarian state along the lines of his own specific brand of “illiberalism”, which he first outlined during his speech at Băile Tuşnad (Tusnádfürdő), Romania on 26 July 2014. In the speech Orbán defined his concept of the “illiberal state” as a specific form of national governance for Hungary and for the wider CEE region, which rejects Western liberal values and is deeply embedded in traditional Christian Democratic values such as the family and the nation (Orbán 2014a). The impact of Orbán’s illiberalism on the Hungarian polity has become ever more visible in the gradual expansion of executive powers at the expense of parliamentary scrutiny, independent judicial supervision of government legislation and the state control of large sections of the media. Orbán has slowly but determinately turned Hungary into a Fidesz state by “unilaterally changing the constitution and replacing key officials in every politically relevant institution” (Kreko and Enyedi 2018, p. 42). An essential feature of this transformation of the Hungarian polity was the gradual expansion of the executive powers of central government at the expense of parliamentary and judiciary scrutiny. This was accompanied by a strategy to undermine the existence of an independent media in favour of government-controlled TV channels and newspapers. The key to this transformation were the introduction of a new constitution in 2011 which strengthened government legislative powers at the expense of legislative scrutiny and judicial supervision by speeding up the implementation of laws and limiting the ability of the Constitutional Court to review legal decisions in key areas such as budgetary matters and taxation (Bos 2021, p. 37; Jakab and Bodnár 2021, p. 64). Under Article 9 (2i) of the constitution the Hungarian president maintains the right to initiate the review of any laws at the Constitutional Court (Parliament Hungary 2021). In practice scrutiny of executive legislation by the Constitutional Court has however in recent years been weakened by the fact that the presidential office has been persistently held by Fidesz party members since 2010 (Pal Schmitt 2010–12; Janos Ader 2012–22 and currently Katalin Novak), who failed to challenge the executive competences of the government. Even more crucial has been the predominantly Fidesz-friendly constellation in the Constitutional Court after 2013, which has facilitated the shift towards the executive under Orban (Pocza 2021, p. 86). This was accompanied by reforms of the electoral system, which paved the way for a shift towards an electoral advantage for Fidesz and the weakening of electoral pluralism. Most of all it granted ethnic Hungarians living in neighbouring countries, such as Slovakia and Romania, the right to vote by postal poll in national elections, while Hungarians living in other countries can only vote by visiting one of the Hungarian consulates or embassies (Toka 2014, p. 4–5). The result has been the inability of the opposition to effectively challenge Fidesz at national elections, even when it formed the broad electoral platform during the 2022 national election campaign.

This “velvet revolution” towards a hybrid system of semi-democratic governance with a clear trend towards authoritarianism has turned Hungary into a prime example of “party state capture”, where the main aim of Fidesz is to tighten its grip on the institutions of the Hungarian state (Agh 2019, p. 95). A central factor in this strategy of “victorious victimization” has been Orbán’s success in presenting himself and Fidesz as the defender of the Hungarian nation against alleged negative external influences (Sükösd 2022). Prime examples are liberal cosmopolitanism and immigration from non-Christian cultures outside Europe represented by the EU and global financial capitalism symbolised by Orbán’s favourite arch enemy, the Hungarian born US businessman George Soros. After more than a decade under the leadership of Orbán, Hungary is leading the way in the CEE region in the general trend towards flawed democracies, where the division of powers between executive, legislative and judicative officially seem to be maintained by existing institutions but in practice are increasingly undermined by insufficient checks and balances, the lack of a pluralist democratic discourse and weak civil societies and expanding executive powers (Dessewffy 2022).

## Managing the Covid pandemic: governance under unprecedented conditions in Germany and Hungary

### Germany: lost in the jungle of multi-level competences?

Germany was hit by the Covid pandemic at a time where the governing majority on the federal level had been substantial as a result of a permanent grand coalition government between the CDU/CSU and the SPD since 2013 under the leadership of Chancellor Angela Merkel. At the same time all regional governments were led by a CDU, CSU or SPD prime minister, with the exception of Baden-Württemberg, where Green prime minister Winfried Kretschmann had governed in coalitions with the SPD and subsequently the CDU since 2011 and also in Thuringia, where Bodo Ramelow has governed as prime minister of Die Linke in a leftist coalition with the SPD and the Greens since 2014. Only Mecklenburg-Vorpommern and Lower Saxony had grand coalition governments when the pandemic emerged in March 2020. In most of the Länder the CDU and the SPD nevertheless also governed with the Bündnis90/Grüne, the FDP or Die Linke, all parties who were in opposition on the federal level. In Bavaria, CSU prime minister Markus Söder had formed a coalition with the Freie Wähler, a party which has its strongholds on the local government level and also entered the Bavarian Landtag after the 2018 regional election on 14 October but has never been represented on the federal level in the Bundestag (Bundesrat 2020). Söder also held the chairmanship of the Ministerpräsidentenkonferenz from 1 October 2019 until 30 September 2020, which made him a prominent figure in the management of the first phase of the pandemic. The governing coalition constellations in each of the 16 Länder is crucial for the overall governance in the German policy because the Basic Law grants the Länder the status of co-decision-makers through the Bundesrat on any legislation which affects their own administrative (Article 84 GG) or financial affairs (Articles 104a and 105 GG) and on legislation which results in changes to the constitution. The latter can only be passed by a two thirds majority in the Bundesrat (Article 79, paragraph 2). Moreover, the implementation of federal legislation also falls into the remit of the Länder administrations (Article 83 GG) (Deutscher Bundestag 2020a). The reform of the federal system in Germany initiated in 2006 has reduced the number of federal laws which require the consent of the majority of the Länder governments in the Bundesrat on the basis of Article 84 GG, while the overall level of consensual laws on financial matters under Articles has been largely maintained (Risse 2007, p. 712). For those laws where the federal government requires the consent of a majority of Länder governments in the Bundesrat, each government coalition needs to find consensus on how to vote on the proposed legislation as each region needs to cast an unanimous vote (Article 51, paragraph 3 GG). In case of internal disagreements within a regional government coalitions on a specific legal issue, the Land in question needs to abstain from the vote (Lang 2003, p. 600; Schmedes 2019, p. 45). The size of the population of the region represented by a government also matters substantially as Article 51, paragraph 2 GG determines that larger regions with a population of more than six million people, such as Bavaria, Baden-Württemberg and North-Rhine Westphalia, cast the maximum number of six votes in the Bundesrat, while smaller regions with less than three million people (Bremen, Hamburg, Mecklenburg-West Pomerania and the Saarland) can only cast three votes.

These details are important as they represent the political background to the management of the Covid pandemic in Germany. The initial phase of the pandemic already showed that the German polity was not sufficiently prepared to manage the unprecedented challenge of a virus pandemic which threatened the health of the entire nation, most significantly of people with chronic illnesses and of the elderly. The national law for the protection of the public against infectious diseases (“Infektionsschutzgesetz”), which had been implemented in 2010, followed the general trend of Germany’s cooperative federalism by limiting the responsibilities of the federal government and delegating the practical crisis management to the administrative level of the Länder (Münch 2020, p. 211). March 2020 witnessed a rapid turn of events when it became clear that what had initially been considered as an isolated infectious outbreak by the then German Health Minister Jens Spahn without any major implications for wider society (Unzicker 2020), would quickly develop into a global virus pandemic. On 11 March 2020 the World Health Organisation (WHO) officially classified the outbreak of the Covid-19 virus as a global pandemic (World Health Organisation 2020). One day later the federal government and the Länder governments met for the first joint Bund-Länder Konferenz (BLK), which was exclusively focused on the management of the emerging pandemic. The BLK on 12 March 2020 was chaired by Chancellor Merkel and represented a new dimension in the informal policy coordination between the federal and the regional level beyond the usual deliberations of the Länder governments in the aftermath of their regularly held Ministerpräsidentenkonferenzen (MPKs). The joint decisions made on the day concentrated on measures to contain the spread of the virus and the setting up of permanent close consultations between federal interior and health ministries and their regional counterparts (Bundesregierung 2020).

The BLK became a regular occurrence during the acute phase of the pandemic and also turned into the main decision-making body on the management of the crisis during final phase of the Merkel government between March 2020 and the autumn of 2021. During this period, Merkel attempted to overcome the divergence of regional perspectives on the management of the pandemic through intensive negotiations with the Länder prime ministers. These often lasted for many hours and frequently revealed significant differences of opinion in how to deal with the crisis. The Corona BLKs once again illustrated that the supposedly cooperative federalism in Germany presents itself as a competitive system when profound regional political interests are at stake. Most significantly the BLK negotiations in 2020/2021 were overshadowed by the run-up to the federal election on 26 September 2021. Between March 2020 and the actual election date, the political consultations in the BLKs were dominated by the intensifying rivalry between CDU leader Armin Laschet and Bavarian prime minister and CSU leader Markus Söder. Both Laschet and Söder aspired to secure the position of official CDU/CSU candidate for the Chancellery for themselves. Söder used his position as chair of the MPK to prominently advocate a cautious approach towards the management of the pandemic. He hence became the main representative of what he branded as team caution (“Team Vorsicht”) and supported Chancellor Merkel in her call for the shutdown of public life, including schools and nurseries, during the first phase of the pandemic from 22 March until 4 May 2020 (Cicero 2022). Merkel and Söder were broadly supported in their restrictive lockdown approach by most of the CDU and the SPD Länder prime ministers, including Winfried Kretschmann, the Green prime minister of Baden-Württemberg. The main dissenters during the initial BLK negotiations were Armin Laschet, the CDU prime minister of North-Rhine Westphalia and Bodo Ramelow, Die Linke prime minister of Thüringia. Laschet publicly criticised the stance of Chancellor Merkel, who had called for the strict obedience of all Länder governments with the recommendations of the Robert Koch Institute (RKI), Germany’s public health institute, whose mandate is to advise the federal government on matters of public health. Laschet emphasised in this respect that he would not strictly follow the recommendations of medical experts but consider the wider “social, human and economic” implications of lockdown policies and the restriction of civil liberties (Detjen 2020). After he had been elected as the new leader of the CDU on 16 January 2021, following the demise of Merkel ally Annegret Kramp-Karrenbauer, Laschet became a persistent advocate of a more relaxed approach towards the pandemic, which was also one of the main dividing lines between him and Söder in their competition for the candidacy as Chancellor (Fahrenholz 2021). The result of these political divisions between the Länder governments on the management of Covid was a growing patchwork of different regulations, which became particularly obvious during the second phase of the pandemic in the autumn/winter of 2021/22.

The first nationwide lockdown in Germany during the initial phase of the pandemic was accompanied by growing concerns about the legitimacy of the decisions made by the BLK and the lack of parliamentary supervision of the executive decisions which had far-reaching implications on public life and on the economy. One of the most prominent critics of the implementation of executive measure on the basis of the BLK negotiations was the Thuringia prime minister Bodo Ramelow from Die Linke. Ramelow rejected Merkel’s claim that she would have the mandate to force the Länder to impose joint measures (“I am not a subordinate authority to the Chancellery”) and called for the greater involvement of the Bundestag in scrutinizing the governance of the pandemic (Der Spiegel 2020). His criticism was echoed by the President of the Bundestag, Wolfgang Schäuble, who on 19 October 2020 addressed the political groups in the federal parliament and called on them to emphasise their role as “the legislative body and the public forum” in the fight against the pandemic and to “avoid the impression that the management of the pandemic would be the exclusive responsibility of executive and legislative” (Deutscher Bundestag 2020b). The lack of participation of the Bundestag was also echoed in the comments made by prominent constitutional experts, such as the former Federal Constitutional Court judge Ferdinand Kirchof (Jungholt 2021). These criticisms reflected the fact that the Bundestag had been marginalised during the first phase of the pandemic in the spring/summer of 2020 in its function to participate in the public debate on strategic political issues and to communicate the reasoning behind legislative decisions to the public. This was less the result of the attempt of the federal and the Länder governments to coordinate their management of the pandemic but due to the combination of the exceptional circumstances, which profoundly restricted normal parliamentary business and the initial hesitation of MPs to submit the BLK deliberations to parliamentary scrutiny. The result was the obvious weakening of the Bundestag’s function as a “discussing and an evaluating parliament” (Marschall 2021, p. 208).

The Merkel government had already initiated the amendment of the “Infektionsschutzgesetz” (IfG) by swiftly pushing a law through parliament and the Bundesrat, which was aimed at protecting the public during an “epidemic situation of nationwide scope” (Bundesgesetzblatt 2020). Under paragraph 5 of the reformed IfG it gave the Bundestag the right to declare a nationwide epidemic situation with a majority of votes, which in turn empowers the federal health ministry to order wide-ranging executive orders aimed at the protection of the public. At the same time the Bundestag has the right to terminate the situation at any time (Ibid). The government yet realised that this had not been sufficient to silence the mounting criticism of the lack of parliamentary scrutiny of the decisions made by the BLK meetings. It hence reacted to these criticisms by introducing a profound revision of the “Infektionsschutzgesetz”, which was rapidly passed by Bundestag and Bundesrat on 19 November 2020. The further revision of paragraph 5 strengthened the involvement of the Bundestag by requiring the federal government to keep parliament constantly informed about the infectious situation, as long as an epidemic situation is officially in place. A new paragraph 28a now lists concrete measures which the federal and the Länder governments can implement to contain a pandemic, which include obligatory face masks, lockdown measures such as curfews and access restrictions in individual areas of public life (Bundesgesundheitsministerium 2020). The second revision of the law was followed by further national lockdown measures from November 2020 until March 2021. The revisions of the IfG continued in 2021 as a result of the lingering Covid pandemic and the emerging availability of vaccinations. The BLK decided to implement a nationwide “emergency brake” on 23 April 2021 which was aimed at avoiding a further nationwide lockdown. The “emergency brake” required cities and regions across Germany to impose contact restrictions if the weekly Covid infection rates exceeded 100 over three subsequent days (Bundesgesundheitsministerium 2021a). The availability of vaccinations against Covid for the majority of the population resulted in a differentiated lockdown strategy in the run-up and following the federal elections in September 2021. In August 2021 the outgoing Merkel government jointly decided on a new “3G” regulation with the Länder governments. “3G” restricted the access to leisure activities, services (such as hairdressers) and visits to hospitals and care homes to individuals who were either vaccinated, had tested positive for Covid during the past six months or had proof of a valid negative test result, as long as individual regions did not decide to temporarily suspend the regulation due to low levels of infections (Bundesregierung 2021).

The final phase of the campaign for the federal election on 26 September was consequently dominated by the question how a new government should approach the ongoing challenge of the Covid pandemic. The FDP and the Alternative für Deutschland (AfD) positioned themselves as critics of the government’s restrictive political approach and called for no further lockdown measures and the gradual phasing out of any restrictions (Die Zeit 2021; Weiland 2021). Armin Laschet, who had emerged as the winner in the competition for the position of official candidate for the Chancellery in the CDU/CSU, came under increasing political pressure with his criticism of the Merkel’s strict management of the pandemic as infection rates soared in his homeland North Rhine-Westphalia, most prominently from his defeated rival Markus Söder (Utz 2021). The resulting weak showing of the CDU/CSU in the election, combined with a less strong result for Bündnis 90/Die Grünen created the opportunity for the SPD to recapture the Chancellery. The new Chancellor Olaf Scholz decided to use the opportunity to form a government coalition with the Greens and the FDP, which is characterised by a relatively large ideological distance between the parties involved. During the final months in office of the outgoing Chancellor Angela Merkel it became increasingly obvious that the coordinative efforts in the context of the BLK meetings has stalled as individual Länder governments, most noticeably those with forthcoming regional elections, failed to abide by the decisions they had previously agreed to. Merkel already included incoming Chancellor Olaf Scholz in the BLK deliberations and managed to push through the temporary general introduction of a nationwide 2G rule, which restricted access to locations to people who were either vaccinated, have proof of recovery from a Covid infection. Merkel’s last BLK on 2 December 2021 was dominated by public disagreements between her and the regional governments on further common measures. Merkel publicly voiced her disappointment that she had been unable to avoid what had become a patchwork of increasingly diverging regional measures in managing the pandemic (Palzer 2021).

During the transition period towards the new government, which took office on 8 December 2021, Merkel reacted to the growing demands of the Länder to be granted the flexibility to determine their own regulations in the management of the virus by introducing a fourth revision of the IfG in November 2021. The new IfG allows the Länder to continue introduce measures to contain the virus based on their individual situation, including the implementation of the stricter “2G”/“2G+” (vaccinated or recovered plus valid negative test result) rule (Bundesgesundheitsministerium 2021b). The new government under Chancellor Olaf Scholz government decided to end the official status of the existence of an epidemic situation in Germany with its governing majority in the Bundestag on 18 November 2022, a fortnight before it came into office. This was confirmed by the Bundesrat a day later. The termination of the status was predominantly the result of demands made by the FDP, who had criticised that the status would result in the growing “dominance of the executive” in Germany (FDP 2021). The new traffic light coalition in the German federal government reflects the diverging positions of the public debate in Germany on the management of the pandemic. These are situated along the dividing line between a more cautious and restrictive approach, represented by the new SPD health minister Karl Lauterbach and his coalition partner Bündnis90/Die Grünen on one side and demands to return towards a new normality with the restoration of individual freedoms, where the protection against Covid becomes mainly an individual responsibility, as advocated by FDP justice minister Marco Buschmann on the other side of the political divide (Focus 2022).

In summary the German case shows that even under exceptional circumstances the foundations of the multi-level system of democratic governance have been maintained and continue to function. In spite of shortcomings in terms of the parliamentary scrutiny of executive decisions, which were jointly decided and implemented by the federal government and the Länder in the context of the numerous BLKs, during the initial phase of the pandemic in 2020, both Bundestag and Bundesrat became sufficiently involved in their role as legislative co-decision-makers. The Bundestag swiftly returned to its role as the main body, which influences and scrutinizes executive decisions, even though more could have been done to communicate this to the general public and to counter the growing impression that parliamentary oversight had become “subordinate” to executive decision-making (Siefken 2022, p. 16). In terms of the role of the Länder, focus on the regular high-level consultations with the federal government show the high adaptability of the federal system towards crisis conditions. The BLK consultations were certainly suitable to achieve relatively swift and at—least until the final phase of the Merkel government—relatively effective joint decisions without sidelining the Bundesrat as the main representative body of Länder interests (Behnke 2021, p. 226; Czypionka and Reiss 2021, p. 300). On the other hand the pandemic once again illustrated a growing problem at the heart of German federalism: the impact of practically constant regional election campaigns during one legislative period and the related tendency of some regional prime ministers to use particularly the BLK deliberations as a platform for their personal political ambitions.

### Hungary: governing by executive decree as the new normality?

The emergence of the Covid pandemic hit Hungary at a time when Fidesz had already come far in consolidating its power base in the Hungarian state. Having defended its two thirds majority at the national elections on 8 April 2018, prime minister Orbán continued encountered the events of the spring of 2020 as a leader with significant executive powers. It quickly became obvious that he would be willing to instrumentalise the pandemic to go even one step further in expanding the powers of the executive at the expense of parliament and the judiciary. The government reacted to the pandemic by initially declaring a “state of danger” on 11 March 2020 in line with Article 53 of the Hungarian Constitution, which was limited to 15 days and would have needed parliamentary approval for an extension. The government did not hesitate to use its broad majority in the Hungarian National Assembly to introduce the “Act XII of 2020” on 30 March 2020 to weaken legislative scrutiny of the executive management of the pandemic. The Act essentially allows the government to declare a “state of danger” during which it can circumvent parliament by ruling by “government decree” (Hungarian Embassy Berlin 2020). In essence this means that the government could suspend or amend laws passed by parliament and introduce additional measures without consulting the National Assembly:

“During the period of the state of danger, in addition to the extraordinary measures and rules laid down in Act CXXVIII of 2011 on disaster management and amending certain related Acts, the Government may, in order to guarantee that life, health, person, property and rights of the citizens are protected, and to guarantee the stability of the national economy, by means of a decree, suspend the application of certain Acts, derogate from the provisions of Acts and take other extraordinary measures” (Section 2, paragraph 1) (Ibid).

Most significantly the Act also included the provision to suspend all by-elections and any national or local referendums during the period when the “state of danger” is in operation (Section 2, paragraph 2 and 3). The only source of potential scrutiny would be the Constitutional Court, which is the only court under the Hungarian constitution which can scrutinize executive government acts. Under Section 5 of the Act the Constitutional Court has the guaranteed right and duty to maintain its normal operations. In practice the current setup of the Constitutional Court has however been substantially influenced by Fidesz, which resulted in government decrees being passed without being challenged by the Court during the repeated declaration of the state of danger (Kovacs 2021). Since its introduction in the Authorization Act, the state of danger was enacted three times by the Hungarian government in reaction to the three waves of the Covid pandemic. The state was officially terminated on 18 June 2020 but followed by a new comprehensive accompanying law, the “Transitional Act”, which introduced a new state of emergency under the title “health crisis situation” which can last up to six months and be continuously extended on the basis of the recommendation of country’s chief medical offices (Pásztor 2020). The health crisis situation was imposed on 18 June 2020 and ultimately extended until 24 May 2022.

Most concerning for the future of Hungarian democracy, the government has repeatedly revised the Act on the “state of danger” with its majority in the National Assembly. With each revision the executive powers of the government during a “state of danger” have been further expanded at the expense of parliament. The first revision on 10 November 2020 granted the government the ability to extend “the applicability of the government decrees under Article 53 (1) and (2) of the Fundamental Law adopted during the period of the state of danger until this Act is repealed” (Hungarian National Law Data Bank 2020). The Second Authorization Act removed the need for parliamentary approval of the extension after 15 days and, which consequently means that the government is able to maintain decrees until it decided to terminate the state of danger, without the need for further approval by parliament. The second revision passed by the Hungarian National Assembly on 22 February 2021 predominantly extended the validity of 70 decrees that would otherwise have terminated (International Commission of Jurists 2022, p. 10). The latest revision passed by the Hungarian National Assembly on 25 May 2022 adopts the act to the current crisis in the Ukraine as a justification for the government to declare another phase of danger for Hungary (Hungarian Helsinki Committee 2022). The Fourth Authorization Act allows the government to declare a “state of emergency” in response to an armed conflict in a neighbouring country, which potentially threatens Hungary’s security. The government reimposed the state of emergency on the basis of the new act and the amendment to the Hungarian constitution on 25 May 2022 (Bos and Kurze 2021). On 27 September 2022 the government used its parliamentary majority to amend the Authorization Act to allow parliament to extend the state of emergency up to a period of 180 days at any time and to renew such permissions repeatedly (Mercédesz 2022).

It has hence become obvious that Orbán has used the Covid pandemic as a pretext for gradually moving towards a new constitutional regime, where the government can use current and future crises to practically rule by decree with only limited parliamentary supervision and obviously non-existent critical oversight by an independent judiciary:

“The state of danger gives Orbán sweeping powers to rule by decree, sidestep parliamentary debate, and suspend laws at short notice with very limited to no judicial oversight (…) It is likely the government will again misuse its powers under the new state of danger to further consolidate control” (Gall 2022).

Most concerning for democratic pluralism in Hungary is the fact that the Act introducing the “health crisis” includes a new Penal Code provision in Section 337, under which any public expression of discontent with the implementation of a health crisis or with any of the measures issued by the government represents a criminal offence which carries the risk of facing a prison sentence of up to three years for the former and up five years for the latter offence. Publicly voiced dissent with the government’s emergency legislation is branded as a “felony”. (Hungarian Embassy Berlin 2020, Section 337). Considering the measures the Fidesz government has introduced since the onset of the Covid pandemic and subsequently under the emerging military conflict in Ukraine, a clear trend towards authoritarian rule becomes visible. This is particularly obvious in the fact that the government has substantially increased the public presence of the police and the military in Hungary and also temporarily closed the borders for non-Hungarian citizens (International Commission of Jurists 2022, p. 11). The fourth election victory for Fidesz since 2010 seems to not only have consolidated the power base for a party which has become a “complex, all-embracing and well-organized economic and social actor” (Agh 2019, p. 91), focused on a personality cult around its leader Viktor Orbán. It has obviously also emboldened Orbán to use the lingering Covid crisis and the most recent Ukraine crisis as means to move towards a new phase in transforming Hungary towards an illiberal one party state with increasingly authoritarian executive powers. Under these circumstances, the Fidesz regime can neither be effectively challenged by a weakened opposition nor by the remaining patches of an independent media and civil society.

## Concluding evaluation: the pandemic as a catalyser for weakening democratic scrutiny?

The comparative analysis presented on the impact of the Covid pandemic on governance in Germany and Hungary summarised in the Table 1 above illustrates that the extraordinary circumstances which followed the outbreak of the virus in the spring of 2020 have had noticeably different effects on polities with a most different constitutional design. In Germany’s federal system of multi-level governance with its constitutionally embedded liberal democracy the basic principles of parliamentary and judicial scrutiny of executive decision-making were maintained, although parliamentary scrutiny was very limited during the initial phase of the pandemic. The need for quick and efficient responses to contain the rapid spreading of the virus and the risk of overburdening medical services did result in unprecedented and sustained efforts to intensify the cooperative aspects of German federalism by initiating the Bund-Länder-Konferenz (BLK) as a new forum for executive decision-making between the federal and the regional governments. This did however not occur at the expense of the established constitutionally embedded institutions in the German polity. Even though the rapid speed of events and the special circumstances of the pandemic made it initially difficult for the Bundestag to keep pace and to fulfill its constitutional role as the legislative body, which scrutinizes and communicates its opinions on executive decision-making, the German polity has not witnessed a fundamental shift towards executive powers at the expense of legislative and independent judicial supervision. This is shown by the fact that not only the Bundestag but also each of the Länder parliaments remained fully involved in debating and passing legislation, as well as by various independent judicial revisions of individual Covid-related legislation by regional courts and the by the Federal Constitutional Court. Most significantly, executive governments on the federal and the regional level never intended to shift the pendulum of the German polity towards the weakening of legislative and judicial revision of legal changes. The German polity has consequently proved to be relatively adaptable and overall resilient to systemic change in response to the challenge of having to manage the containment of the pandemic.

**Table 1:** Covid-related Governance Changes

Germany	Hungary
Strengthening of coordination of executive governance between the federal and the Länder level: March 2020–2021 regular *Bund-Länder Konferenzen (BMK) *consultations on the coordination of protective measures against the pandemic March-May 2020 BMK determines **first nationwide lockdown** which closes majority of shops, bars and restaurants, nurseries, schools and hospitals and prohibits public events (limited regional and federal parliamentary scrutiny) November 2020 Revision of the *Infektionsschutzgesetz (IfSG)* introduced in 2010, granting the Länder the right to impose wide-ranging restrictions of public life under pandemic conditions (full parliamentary scrutiny on the federal and regional levels) December 2020–Mai 2021**s nationwide lockdown** with regional variations (limited scrutiny by the Bundestag and the Länder parliaments) April 2021–December 2021 further revisions to the **IfSG**: **“emergency brake”** requires Länder and municipalities to introduce restrictions to the access to social amenities and public events by requiring tests and proof of vaccination or previous Covid infections **(“3G”/“2G”+) **(full parliamentary scrutiny on the federal and regional levels)	Weakening of legislative and judicial scrutiny by introducing decree powers for central executive government: March-June 2020 *Coronavirus Containment Act * introduces “state of danger” (unlimited) June 2020 *Transitional Act *introduces “health crisis situation” (limited to six months, renewable) May 2022 *Fourth Authorization Act* introduces “state of emergency” in response to an armed conflict in the neigbourhood (180 days, renewable with parliamentary consent)

Although Covid did not act as an independent variable towards constitutional drift in favour of the executive in the German polity, the pandemic has nevertheless revealed deficiencies in the political culture of the contemporary Germany, which relate most of all to the lack of controversial public debates in parliament and in the media during the initial phase of the pandemic (Merkel 2020). The German Ethics Council warns in its assessment of the complexity of the Covid crisis that as a result “public trust in the German state as a democracy, constitutional state and federal state has suffered during the pandemic.” (Deutscher Ethikrat 2022, p. 23). It will be crucial for German political elites to realise this and to draw conclusions on how to approach future crises if they do not want to risk long-term damage to the socially embedded legitimacy of the German polity. The fact that two thirds of voters in Germany recently expressed the opinion in a poll conducted by the Allensbach Institute that they would live in a “facade democracy” should be a warning shot to regional and federal decision-makers. (Die Welt 2022).

In the case of Hungary, the pandemic has acted as a catalyst for the intensification of democratic backsliding and the weakening of the foundations of the already fragile democratic state. The Fidesz government under the leadership of prime minister Orbán has used the pandemic as a tool to at least temporarily remove existing constraints on its already substantial executive powers by instilling a new regime of governing by decree during times of crises. The unprecedented challenge of the pandemic has in this respect acted as an independent variable which facilitated the government’s attempts to weaken the checks and balances in the Hungarian polity even further (Löblová, Rone and Borbáth 2021, p. 415 and 424). The fact that the constitutional changes which resulted from the Covid pandemic have been expanded further during the more recent Ukraine crisis, shows however that Orbán is willing to instrumentalise any crisis for his political agenda to consolidate a hybrid illiberal regime of “competitive authoritarianism” with Fidesz as the new permanent governance agent (Marantz 2022). The obvious drift of Hungary under Fidesz from a flawed democracy towards a hybrid regime with increasingly obvious tendencies towards authoritarianism poses a major challenge for the liberal democracies in the EU’s core. The EU’s patchy response to the trend towards illiberalism in the Central-Eastern periphery, which currently lacks a more coherent strategy and focuses predominantly on individual issues, such as the misallocation of EU funds, will be hardly sufficient to bring the trend towards democratic backsliding in Hungary, Poland and other countries in the region to a halt. As long as EU membership remains attractive for the region on the basis of the embeddedness in the Single Market and in particularly the German export chain, Hungary and the defective democracies in its CEE neighbourhood will remain “externally constrained hybrid regimes” (Bozoki and Hegedüs 2021). This offers some scope for influencing domestic developments in Hungary by reminding the Hungarian government of their responsibility to maintain the foundations of the democratic state under the EU membership criteria.

## References

[CR1] Agh, Attila. 2016. The increasing core-periphery divide and new member states: diverging from the European Union’s mainstream developments. In *Core-periphery relations in the European Union*, ed. José M. Magone, Brigid Laffan, and Christian Schweiger, 117–129. Oxon: Routledge.

[CR2] Agh, Attila. 2019. *Declining democracy in east-central europe: the divide in the EU and emerging hard populism*. Cheltenham: Edward Elgar.

[CR3] Bache, Ian. 2015. Cohesion policy: a new direction for new times? In *Policy-making in the European Union*, 243–262. Oxford: University Press.

[CR4] Behnke, Nathalie. 2021. Föderalismus in der (Corona)Krise? – Föderale Funtionen, Kompetenzen und Entscheidungsprozesse. In *Corona Pandemie und Krise*, ed. Anne-Sophie Friedel, Bundeszentrale für Politische Bildung, 215–229. Bonn: Bundeszentrale für Politische Bildung.

[CR5] Bolleyer, Nicole and Orsolya Salát. 2021. Parliaments in times of crisis: COVID-19, populism and executive dominance. *West European Politics* 44(5–6):1103–1128.

[CR6] Bos, Ellen. 2021. Politisches System und Demokratientwicklung in Ungarn: Funktionsdefizite und Instrumentalisierung demokratischer Verfahren durch die Regierungsparteien. In *Das politische System Ungarns: Nationale Demokratieentwicklung, Orbán und die EU*, ed. Ellen Bos, Astrid Lorenz, 25–56. Wiesbaden: Springer.

[CR7] Bos, Ellen, and Kristina Kurze. 2021. On the adaption of the EU’s new rule of law conditionality mechanism: the COVID-19 crisis as a window of opportunity. *L’Europe En Formation* 392(1):87–108.

[CR8] Bozoki, Andras, and Daniel Hegedüs. 2021. *The rise of authoritarianism in the European Union: a hybrid regime in Hungary*. Abingdon: Routledge.

[CR9] Bulmer, Simon, and William E. Paterson. 2013. Germany as the EU’s reluctant hegemon? Of economic strength and political constraints. *Journal of European Public Policy* 20:1387–1405.

[CR10] Bulmer, Simon, and William E. Paterson. 2019. *Germany and the European Union: Europe’s Reluctant Hegemon?* London: Red Globe Press.

[CR11] Bundesgesetzblatt. 2020. Gesetz zum Schutz der Bevölkerung bei einer epidemischen Lage von nationaler Tragweite. 27 March. https://www.bgbl.de/xaver/bgbl/start.xav?startbk=Bundesanzeiger_BGBl&start=//*%5b@attr_id=%27bgbl120s0587.pdf%27%5d#__bgbl__%2F%2F*%5B%40attr_id%3D%27bgbl120s0587.pdf%27%5D__1656494535026. Accessed 1 Nov 2022.

[CR12] Bundesgesundheitsministerium. 2020. Drittes Gesetz zum Schutz der Bevölkerung bei einer epidemischen Lage von nationaler Tragweite. 18 November. https://www.bundesgesundheitsministerium.de/fileadmin/Dateien/3_Downloads/Gesetze_und_Verordnungen/GuV/B/3._BevSchG_BGBl.pdf. Accessed 1 Nov 2022.

[CR13] Bundesgesundheitsministerium. 2021a. Viertes Gesetz zum Schutz der Bevölkerung bei einer epidemischen Lage von nationaler Tragweite. 22 April 2021. https://www.bundesgesundheitsministerium.de/fileadmin/Dateien/3_Downloads/Gesetze_und_Verordnungen/GuV/B/4_BevSchG_BGBL.pdf. Accessed 1 Nov 2022.

[CR14] Bundesgesundheitsministerium. 2021b. Gesetz zur Änderung des Infektionsschutzgesetzes und weiterer Gesetze anlässlich der Aufhebung der Feststellung der epidemischen Lage von nationaler Tragweite. 22 November 2021. https://www.bundesgesundheitsministerium.de/fileadmin/Dateien/3_Downloads/Gesetze_und_Verordnungen/GuV/I/IfSG-Aend_Bgbl_231121.pdf. Accessed 1 Nov 2022.

[CR15] Bundesrat. 2020. Zusammensetzung des Bundesrates vom 4. März 2020 bis 17. Mai 2021. https://www.bundesrat.de/SharedDocs/bilder/DE/galerien/stimmenverteilung-br/stimmenverteilung-br/20200304-zusammensetzung-br.jpg?__blob=poster&v=7. Accessed 1 Nov 2022.

[CR17] Bundesregierung. 2020. Besprechung der Bundeskanzlerin mit den Regierungschefinnen und Regierungschefs der Länder. 12 March. https://www.bundesregierung.de/breg-de/themen/coronavirus/beschluss-zu-corona-1730292. Accessed 1 Nov 2022.

[CR18] Bundesregierung. 2021. Bund-Länder Beratung Corona: 3G-Regel. https://www.bundesregierung.de/breg-de/aktuelles/bund-laender-beratung-corona-1949606. Accessed 1 Nov 2022.

[CR19] Cicero. 2020. Markus Söder: Team Vorsicht und Team Augenmaß. 17 January. https://www.cicero.de/innenpolitik/markus-soder-team-vorsicht-und-team-augenmass-corona-omikron-impfpflicht-querdenker-lockdown. Accessed 1 Nov 2022.

[CR20] Czypionka, Thomas, and Miriam Reiss. 2021. Three approaches to handling the Covid-19 crisis in federal countries: Germany, Austria and Switzerland. In *The comparative politics and policy of COVID-19*, ed. Scott L. Greer, et al., 295–319. University of Michigan Press.

[CR21] Der Spiegel. 2020. Ramelow erinnert Ministerpräsidenten an die “Grenzen ihrer Kompetenzen”. *Der Spiegel*. 27 October. https://www.spiegel.de/politik/deutschland/corona-krise-bodo-ramelow-kritisiert-entscheidungen-von-bund-und-laendern-a-085e84e6-f40a-4826-b19f-39bb4979e249. Accessed 1 Nov 2022.

[CR23] Dessewffy, Tibor. 2022. Orban’s Integrity Office: Everything is legal but nothing is democratic. 30 September. https://ecfr.eu/article/orbans-integrity-office-everything-is-legal-but-nothing-is-democratic/. Accessed 1 Nov 2022.

[CR22] Detjen, Stephan. 2020. Lockerung der Coronavirus-Maßnahmen Laschet (CDU): Mir sagen nicht Virologen, was ich zu entscheiden habe. 19 April. https://www.deutschlandfunk.de/lockerung-der-coronavirus-massnahmen-laschet-cdu-mir-sagen-100.html. Accessed 1 Nov 2022.

[CR24] Deutscher Bundestag. 2020a. Grundgesetz für die Bundesrepublik Deutschland. https://www.bundestag.de/gg. Accessed 1 Nov 2022.

[CR25] Deutscher Bundestag. 2020b. Schäuble: Rolle als Ge-setz-geber in der Pan-de-mie-be-kämp-fung deutlich machen. https://www.bundestag.de/dokumente/textarchiv/2020/kw43-parlamentsbeteiligung-corona-800010. Accessed 1 Nov 2022.

[CR26] Deutscher Ethikrat. 2022. Vulnerabilität und Resilienz in der Krise – Ethische Kriterien für Entscheidungen in einer Pandemie: Stellungnahme. 4 April. https://www.ethikrat.org/fileadmin/Publikationen/Stellungnahmen/deutsch/stellungnahme-vulnerabilitaet-und-resilienz-in-der-krise.pdf. Accessed 1 Nov 2022.

[CR27] Die Welt. 2022. Allensbach Institut: Fast ein Drittel der Bundesbürger glaubt in “Scheindemokratie” zu leben. 11 April. https://www.welt.de/politik/deutschland/article238105613/Umfrage-Allensbach-Fast-ein-Drittel-der-Bundesbuerger-glaubt-in-Scheindemokratie-zu-leben.html. Accessed 1 Nov 2022.

[CR28] Die Zeit. 2021. Wahlkampf: AfD kritisiert Energie- und Corona-Politik. *Die Zeit*. 24 September. https://www.zeit.de/news/2021-09/24/wahlkampf-afd-kritisiert-energie-und-corona-politik?utm_referrer=https%3A%2F%2Fwww.google.com%2F. Accessed 1 Nov 2022.

[CR30] Economist Intelligence Unit. 2002. Democracy index 2021: the China challenge. https://www.eiu.com/n/campaigns/democracy-index-2021. Accessed 1 Nov 2022.

[CR29] Engler, Sarah, et al, 2021. Democracy in times of the pandemic: explaining the variation of COVID-19 policies across European democracies. *West European Politics* 44(5–6):1077–1102.

[CR31] Fahrenholz, Peter. 2021. Was Söder weiß – und Laschet nicht glaubt. *Süddeutsche Zeitung*. 28 July. https://www.sueddeutsche.de/meinung/corona-politik-markus-soeder-armin-laschet-csu-cdu-reiserueckkehrer-1.5365800. Accessed 1 Nov 2022.

[CR32] Farkas, Beáta. 2018. Central European relations in turbulent times. In *Central and eastern Europe in the EU*, ed. Christian Schweiger, Anna Visvizi, 57–73. Abingdon: Routledge.

[CR33] Farkas, Beata, and Andras Mate-Toth. 2018. A rift in European integration? Neglected shadows of the central and eastern European transformation. In *Central and eastern Europe in the EU: challenges and perspectives under crisis conditions*, ed. Christian Schweiger, Anna Visvizi, 25–43. London, New York: Routledge.

[CR34] FDP. 2021. Corona-Notlage: Der Gesundheitsnotstand muss jetzt enden. 27 October. https://www.fdp.de/der-gesundheitsnotstand-muss-jetzt-enden.Accessed. Accessed 1 Nov 2022.

[CR35] Focus. 2022. Grüne machen FDP Druck “Einige suchen gezielt Streit”: Ampel ringt um neuen Corona-Fahrplan für den Herbst. 5 June. https://www.focus.de/gesundheit/coronavirus/gruene-machen-fdp-druck-einige-suchen-gezielt-streit-ampel-ringt-um-neuen-corona-fahrplan-fuer-den-herbst_id_107945063.html. Accessed 1 Nov 2022.

[CR36] Foundation Robert Schuman. 2010. The Fidesz wins the 2/3 majority in the Hungarian Parliament. 26 April. https://www.robert-schuman.eu/en/eem/1016-the-fidesz-wins-the-2-3-majority-in-the-hungarian-parliament. Accessed 1 Nov 2022.

[CR37] Gall, Lydia. 2022. Hungary’s new “state of danger”: Orban Instrumentalizes Ukraine war to further consolidate power. 8 June. https://www.hrw.org/news/2022/06/08/hungarys-new-state-danger. Accessed 1 Nov 2022.

[CR38] Hegele, Yvonne, and Nathalie Behnke. 2013. Die Landesministerkonferenzen und der Bund – Kooperativer Föderalismus im Schatten der Politikverflechtung. *Politische Vierteljahresschrift* 54(1):21–49.

[CR39] Hegele, Yvonne, and Johanna Schnabel. 2021. Federalism and the management of the COVID-19 crisis: centralisation, decentralisation and (non-)coordination. *West European Politics* 44(5–6):1052–1076.

[CR40] Holtmann, Everhard. 1999. Bundesländer. In *Handbuch zur Deutschen Einheit 1949–1989–1999*, ed. Werner Weidenfeld, Karl-Rudolf Korte, 86–99. Bonn: Bundeszentrale für Politische Bildung.

[CR41] Hoyer, Wolfgang. 2001. National decision-making structures for German European policy. In *Germany’s new foreign policy: decision-making in an interdependent world*, ed. W.D. Eberwein, K. Kaiser, 157–170. Basingstoke, New York: Palgrave.

[CR42] Hungarian Embassy Berlin. 2020on. Act XII of 2020 on the Containment of Corona Virus. https://berlin.mfa.gov.hu/assets/77/49/43/cc3672166e33b2cf015ce4371aeedf19417c2710.pdf. Accessed 1 Nov 2022.

[CR44] Hungarian Helsinki Committee. 2022. New “authorization act” removes parliamentary oversight over emergency government decrees. 10 June. https://helsinki.hu/en/new-authorization-act-removes-parliamentary-oversight-over-emergency-government-decrees/. Accessed 1 Nov 2022.

[CR45] Hungarian National Law Data Bank. 2020on. Act CIX of 2020 on the containment of the second wave of the coronavirus pandemic. 11 November. https://njt.hu/translation/J2020T0109P_20201111_FIN.pdf. Accessed 1 Nov 2022.

[CR46] Hungary Today. 2022. Election 2022—election results become final. 19 April. https://hungarytoday.hu/election-2022-election-results-become-final-fidesz-mi-hazank/. Accessed 1 Nov 2022.

[CR47] International Commission of Jurists. 2022. A facade of legality: COVID-19 and the exploitation of emergency powers in Hungary. https://www.icj.org/wp-content/uploads/2022/02/Hungary-A-Facade-of-Legality-legal-briefing-2022-ENG.pdf. Accessed 1 Nov 2022.

[CR48] Jakab, András, and Eszter Bodnár. 2021. Agonie eines jungen Verfassungstaates.Die ungarische Verfassung 1989 bis 2019. In *Das politische System Ungarns: Nationale Demokratieentwicklung, Orbán und die EU*, ed. Ellen Bos, Astrid Lorenz, 57–74. Wiesbaden: Springer.

[CR50] Jeffery, Charlie, and Niccole M. Pamphilis. 2016. The myth and paradox of “uniform living conditions” in the German federal system. *German Politics* 25(2):176–192.

[CR49] Jeffery, Charlie, and Carolyn Rowe. 2014. The reform of German federalism. In *Developments in German politics*, ed. Stephen Padget, William E. Paterson, and Reimut Zohlnhöfer, 35–56. Basingstoke: PalgraveMacMillan.

[CR51] Jungholt, Thorsten. 2021. Ex-Verfassungsrichter Kirchof: “Man kann eine Gesellschaft auch zu Tode schützen“. 2 April. https://www.welt.de/politik/deutschland/plus229606515/Ferdinand-Kirchhof-Man-kann-eine-Gesellschaft-auch-zu-Tode-schuetzen.html. Accessed 1 Nov 2022.

[CR52] Kovacs, Kriszta. 2021. Hungary and the pandemic: a pretext for expanding power. 11 March. https://verfassungsblog.de/hungary-and-the-pandemic-a-pretext-for-expanding-power/. Accessed 1 Nov 2022.

[CR53] Kreko, Peter, and Zsolt Enyedi. 2018. Explaining Eastern Europe: Orbán’s Laboratory of Illiberalism. *Journal of Democracy* 29(3):39–51.

[CR54] Kundnani, Hans. 2014. *The paradox of German power*. London: Hurst.

[CR55] Lang, Joachim. 2003. Zur Frage uneinheitlichen Stimmverhaltens im Bundesrat. *Zeitschrift für Parlamentsfragen* 34(3):596–605.

[CR56] Lendvai, Paul. 2017. *Orbán: Europe’s new strongman*. Hurst&Co.

[CR58] Löblová, Olga, Julia Rone, and Endre Borbáth. 2021. Covid-19 in central and eastern Europe: focus on Czechia, Hungary and Bulgaria. In *The comparative politics and policy of COVID-19*, ed. Scott L. Greer, et al., 413–435. University of Michigan Press.

[CR57] Lorenz, Astrid, and Ellen Bos. 2021. Das politische System Ungarns zwischen Parteienwettbewerb und strukturellen Zwängen: Innenpolitische Polarisierung trotz konstanter Verhaltensmuster und Konsens in den Grundlinien der Außen- und Wirtschaftspolitik. In *Das politische System Ungarns: Nationale Demokratieentwicklung, Orbán und die EU*, ed. Ellen Bos, Astrid Lorenz, 1–24. Wiesbaden: Springer.

[CR59] Marantz, Andrew. 2022. Does Hungary offer a glimpse of our authoritarian future?. *The New Yorker*. 4 July. https://www.newyorker.com/magazine/2022/07/04/does-hungary-offer-a-glimpse-of-our-authoritarian-future. Accessed 1 Nov 2022.

[CR60] Marschall, Stefan. 2021. Parlamente in der Krise? Der Deutsche Parlamentarismus. In *Corona Pandemie und Krise*, ed. Bundeszentrale für Politische Bildung, Anne-Sophie Friedel, et al., 203–214. Bonn: Bundeszentrale für Politische Bildung.

[CR61] Maskarinec, Pavel, and Jakub Charvát. 2022. On the way to limited competitiveness: political consequences of the 2011 electoral reform in Hungary. *Swiss Political Review*10.1111/spsr.12535.

[CR62] Mercédesz, Hetzmann. State of Emergency in Hungary may be extended by 180s whenever from November. 28 September. https://dailynewshungary.com/state-of-emergency-in-hungary-may-be-extended-by-180-days-whenever-from-november/. Accessed 1 Nov 2022.

[CR63] Merkel, Wolfgang. 2020. Wer regiert in der Krise? Demokratie in Zeiten der Pandemie. *WSI Mitteilungen* 73(6):445–453.

[CR64] Münch, Ursula. 2020. Wenn dem Bundesstaat die Stunde der Exekutive schlägt: der deutsche (Exekutiv‑)Föderalismus in Zeiten der Coronakrise. In *Jahrbuch des Föderalismus 2020: Föderalismus, Subsidiarität und Regionen in Europa*, ed. Europäisches Zentrum für Föderalismus-Forschung Tübingen, 209–225. Baden-Baden: Nomos.

[CR65] Nikolai, Rita. 2020. Schulpolitik im Deutschen Föderalismus. In *Reformbaustelle Bundesstaat*, ed. Felix Knüpling, Mario Kölling, Sabine Kropp, and Henrik Scheller, 315–332. München: Springer.

[CR66] Nyikos, Györgi. 2021. Regionalpolitik in Ungarn: EU-Einflüsse und die (re-) zentralisierte Verfolung nationaler Prioritäten. In *Das politische System Ungarns: Nationale Demokratieentwicklung, Orbán und die EU*, ed. Ellen Bos, Astrid Lorenz, 247–266. Wiesbaden: Springer.

[CR67] Orbán, Viktor. 2014a. Speech at Baile Tusnad (Tusnádfürdö). 26 July. https://budapestbeacon.com/full-text-of-viktor-Orbáns-speech-at-baile-tusnad-tusnadfurdo-of-26-july-2014/. Accessed 1 Nov 2022.

[CR68] Palzer, Kerstin. 2021. Bund-Länder-Gipfel. Warum Merkel nicht zufrieden ist. *Tagesschau.de. *1 December. https://www.tagesschau.de/inland/bund-laender-beratungen-coronavirus-105.html. Accessed 1 Nov 2022.

[CR69] Parliament of Hungary. 2021. The Fundamental Law of Hungary. https://www.parlament.hu/documents/125505/138409/Fundamental+law/73811993-c377-428d-9808-ee03d6fb8178. Accessed 1 Nov 2022.

[CR70] Pásztor, Emese. 2020. Rule of law “light”: The “state of medical emergency” in Hungary. 18 September. https://www.boell.de/en/2020/09/18/rule-law-light-state-medical-emergency-hungary. Accessed 1 Nov 2022.

[CR71] Paterson, William. 2011. The reluctant hegemon? Germany moves centre stage in the EU. *Journal of Common Market Studies* 49:57–75.

[CR72] Pocza, Kálmán. 2021. Umkämpftes Terrain: Politik und Verfassungsgericht in Ungarn seit 1990. In *Das politische System Ungarns: Nationale Demokratieentwicklung, Orbán und die EU*, ed. Ellen Bos, Astrid Lorenz, 247–266. Wiesbaden: Springer.

[CR73] Pogatsa, Zoltan. 2009. Hungary: from star transition student to backsliding member state. *Journal of Contemporary European Research* 5(4):597–613.

[CR74] Ragnitz, Joachim, and Marcel Thum. 2019. Gleichwertig, nicht gleich. Zur Debatte um die “Gleichwertigkeit der Lebensverhältnisse”. *Aus Politik und Zeitgeschichte* 69(46):13–18.

[CR75] Risse, Horst. 2007. Zur Entwicklung der Zustimmungsbedürftigkeit von Bundesgesetzen nach der Föderalismusreform 2006. *Zeitschrift für Parlamentsfragen* 38(4):707–712.

[CR78] Scharpf, Fritz W. 1999. Föderale Politikverflechtung: Was muß man ertragen – was kann man ändern?. MPIfG Working Paper 99/3. https://pure.mpg.de/rest/items/item_1235397/component/file_2366365/content. Accessed 1 Nov 2022.

[CR76] Scharpf, Fritz W. 1988. The joint decision-trap: lessons from German federalism and European integration. *Public Administration* 66(3):239–278.

[CR77] Scheller, Henrik. 2020. Zur Reform des Bildungsföderalismus: Kooperative Unitarisierung durch Institutionenbildung? In *Reformbaustelle Bundesstaat*, ed. Felix Knüpling, Mario Kölling, Sabine Kropp, and Henrik Scheller, 333–363. München: Springer.

[CR79] Schmedes, Hans-Jörg. 2019. *Der Bundesrat in der Parteiendemokratie: Aufgaben, Struktur und Wirkung der Länderkammer im föderalen Gefüge*. Berlin: Nomos.

[CR80] Siefken, Sven T. 2022. The Bundestag in the pandemic year 2020/21—continuity and challenges in the Covid-19 crisis. *German Politics*10.1080/09644008.2021.2024806.

[CR81] Sturm, Roland. 2013. Zusammenarbeit im deutschen Föderalismus. *Bpb Informationen zur politischen Bildung/izpb*. https://www.bpb.de/shop/zeitschriften/izpb/159339/zusammenarbeit-im-deutschen-foederalismus/. Accessed 1 Nov 2022.

[CR82] Sükösd, Miklos. 2022. Victorious victimization: Orbán the orator—deep securitization and state populism in Hungary’s propaganda state. In *Populist rhetorics: case studies and a minimalist definition*, ed. Christian Kock, Lisa Villadsen, 165–186. Basingstoke: PalgraveMacmillan.

[CR83] Toka, Gábor. 2014. Constitutional principles and electoral democracy in Hungary. In *Constitution building in consolidated democracies: a new beginning or decay of a political system?*, ed. Ellen Bos, Kálman Pocza, 1–14. Baden-Baden: Nomos.

[CR84] Unzicker, Alexander. 2020. Corona: Die unerträgliche Inkompetenz des Jens Spahn. *Telepolis*. 18 April. https://www.heise.de/tp/features/Corona-Die-unertraegliche-Inkompetenz-des-Jens-Spahn-4705173.html. Accessed 1 Nov 2022.

[CR85] Utz, Tobias. 2021. Delta-Variante: Armin Laschet stellt Corona-Prognose auf – und erntet dafür Hohn und Spott. *Frankfurter *Rundschau. 29 June. https://www.fr.de/panorama/corona-armin-laschet-delta-variante-inzidenz-deutschland-coronavirus-kanzlerkandidat-union-bundestagswahl-90826105.html. Accessed 1 Nov 2022.

[CR86] Weiland, Severin. 2021. FDP in der Coronakrise: Trip in die Wirklichkeit. *Der Spiegel*. 1 December. https://www.spiegel.de/politik/deutschland/fdp-und-corona-und-pandemie-die-lange-reise-in-die-wirklichkeit-a-0e85497f-f34e-4a88-9e0c-665b9e915a90. Accessed 1 Nov 2022.

[CR87] World Health Organisation. 2020. WHO Director-General’s opening remarks at the media briefing on COVID-19. 11 March. https://www.who.int/director-general/speeches/detail/who-director-general-s-opening-remarks-at-the-media-briefing-on-covid-19---11-march-2020. Accessed 1 Nov 2022.

